# TROP2 expression in head and neck squamous cell carcinoma: association with the tumor immune microenvironment and clinical outcome

**DOI:** 10.3389/fonc.2026.1901872

**Published:** 2026-08-06

**Authors:** Lukas A. Brust, Jan Philipp Kühn, Sandrina Körner, Moritz Knebel, Felix L. Braun, Ala-Addean Mustapha, Silke Wemmert, Bernhard Schick, Mathias Wagner, Martin Ertz, Yoo-Jin Kim, Maximilian Linxweiler

**Affiliations:** 1 Department of Otorhinolaryngology, Head and Neck Surgery, Saarland University, Homburg, Germany; 2 Department of General and Surgical Pathology; Saarland University, Homburg/Saar, Germany

**Keywords:** HNSCC, IHC, TCGA, TME, TROP2, ICI

## Abstract

**Introduction:**

TROP2 is a transmembrane glycoprotein implicated in tumor progression and immune regulation across epithelial malignancies and serves as a therapeutic target for antibody -drug conjugates. Its role in the tumor immune microenvironment of head and neck squamous cell carcinoma (HNSCC), particularly in the context of PD-1 blockade, remains insufficiently defined.

**Methods:**

TROP2 protein expression was assessed by immunohistochemistry in 47 patients with HNSCC treated with anti -PD-1 therapy, including primary tumors and, in 27 cases, matched recurrent and/or metastatic lesions. Expression was quantified using the immunoreactive score. Tumor-infiltrating immune cells (CD3, CD8, CD4, FOXP3, CCR4, and CD163) were analyzed in intra- and peritumoral compartments. In parallel, transcriptomic data from The Cancer Genome Atlas (TCGA; n = 483) were evaluated to explore associations between TROP2 mRNA expression and immune infiltration signatures.

**Results:**

TROP2 expression was detected in 95% of tumors, with more than 80% showing moderate to high levels. Higher TROP2 expression was significantly associated with lower T status and showed a trend toward absence of distant metastasis but was not associated with nodal status, UICC stage, disease setting, or response to PD-1 blockade. Increased intratumoral CCR4+ and CD3+ T-cell infiltration correlated with improved survival. TCGA analysis demonstrated significant associations between TROP2 expression and multiple immune cell populations, including CD8+ and CD4+ T cells, Th2 cells, dendritic cells, macrophages, and TGF-β -related signatures.

**Discussion:**

These findings indicate that TROP2 is broadly expressed in HNSCC and linked to distinct immune microenvironment features, supporting its potential as a therapeutic target.

## Introduction

Head and neck squamous cell carcinoma (HNSCC) is the sixth most common malignancy worldwide and remains associated with substantial morbidity and mortality despite advances in multimodal therapy (1, 2). Established risk factors include tobacco use, alcohol consumption, and infection with high-risk human papillomavirus, particularly in oropharyngeal cancer (3). Immune checkpoint inhibition targeting the PD-1/PD-L1 axis has reshaped the therapeutic landscape in recurrent or metastatic HNSCC (4). Phase III trials such as CheckMate 141, KEYNOTE-048 and KEYNOTE-040 demonstrated improved survival with nivolumab and pembrolizumab, respectively, leading to their approval in this setting (5–7). More recently, the phase III KEYNOTE-689 trial demonstrated that perioperative pembrolizumab significantly improves outcomes in patients with resectable, locally advanced HNSCC, extending the role of PD-1 blockade into earlier disease stages (8–10). However, only a subset of patients derive durable benefit, and predictive biomarkers beyond PD-L1 expression on tumor and/or immune cells remain insufficient.

Trophoblast cell surface antigen 2 (TROP2), encoded by *TACSTD2*, is a transmembrane glycoprotein involved in epithelial cell proliferation, adhesion, and signal transduction and calcium signaling (11). TROP2 overexpression has been reported in multiple epithelial malignancies and is frequently associated with aggressive tumor biology and poor prognosis (12, 13). Mechanistically, TROP2 has been linked to MAPK/ERK, JAK/STAT and EGFR signaling, epithelial–mesenchymal transition, and stemness-associated pathways (11, 13–15). Its clinical relevance is underscored by the development of FDA and EMA approved antibody–drug conjugates (ADC) such as Sacituzumab govitecan which links a humanized anti-TROP2 monoclonal antibody to the topoisomerase-I inhibitor SN-38, enabling targeted cytotoxic delivery in metastatic triple-negative breast cancer and metastatic hormone receptor-positive/HER2-negative breast cancer (16–19).

Beyond its role in tumor cell–intrinsic signaling, emerging evidence suggests that TROP2 may influence the tumor microenvironment (TME). HNSCC is characterized by a highly heterogeneous immune landscape, with tumor-infiltrating lymphocytes, regulatory T cells, and macrophage subsets contributing to immune evasion and therapeutic response (20–22). While CD8+ T-cell density and PD-L1 expression have been studied extensively, the relationship between TROP2 expression and immune cell composition in HNSCC remains poorly defined.

Given the increasing importance of immunotherapy in HNSCC and the therapeutic targeting of TROP2 in solid tumors, a better understanding of TROP2 expression patterns, their association with clinicopathologic parameters, and their interaction with the immune microenvironment is warranted. In this study, we performed a comprehensive immunohistochemical and transcriptomic analysis of TROP2 in primary and recurrent/metastatic HNSCC, with particular focus on immune contexture and outcomes under PD-1 blockade.

## Materials and methods

### Patient cohort and study design

This retrospective study included 47 patients with histologically confirmed HNSCCs treated at Saarland University Medical Center (Homburg/Saar, Germany) between 2016 and 2024. Clinical data, including demographic characteristics, tumor localization, TNM classification, UICC stage, treatment modalities, and follow-up information, were obtained from institutional medical records (summarized in **Table 1**).

**Table 1:** Clinical and clinicopathological characteristics of the HNSCC patient cohort classified according to the UICC TNM staging system (8th edition).

Characteristic	Category	HNSCC patients (n = 47)
No. of patients		47
Mean Age [years]		64.4
Sex	male	40 (85.1%)
	female	7 (14.9%)
Localization	Oral cavity	8 (17.0%)
	Oropharynx	18 (38.3%)
	Larynx	12 (25.5%)
	Hypopharynx	9 (19.1%)
Immunotherapy	Pembrolizumab	29 (61.7%)
	Nivolumab	18 (38.3%)
HPV Status	Negative	42 (89.4%)
	Positive	5 (10.6%)
T* stage	1	4 (8.5%)
	2	10 (21.3%)
	3	14 (29.8%)
	4	19 (40.4%)
N* stage	0	13 (27.7%)
	1	6 (12.8%)
	2	19 (40.4%)
	3	9 (19.1%)
M* stage	0	33 (70.2%)
	1	14 (29.8%)
UICC* Stage	I	1 (2.1%)
	II	2 (4.2%)
	III	4 (8.5%)
	IV	40 (85.1%)
Response to immunotherapy	CR	3 (6.4%)
	PR	8 (17.0%)
	SD	13 (27.7%)
	PD	23 (48.9%)

All patients provided written informed consent for the scientific use of their tissue samples and clinical data. The study was approved by the Saarland Ethics Review Board, Saarland, Germany (reference number 218/10), and conducted in accordance with the Declaration of Helsinki and applicable regulatory guidelines.

Tumor staging was performed according to the 8th TNM classification system of the Union for International Cancer Control (UICC). The study cohort included patients with recurrent and/or metastatic (R/M) HNSCC, either presenting with primary metastatic disease at initial diagnosis or developing recurrence or progression after prior therapy, all of whom received immune checkpoint inhibitors in the palliative setting. In one exceptional case with initial UICC stage I disease, subsequent clinical circumstances led to an individualized palliative treatment approach with primary immunotherapy due to a history of multiple prior malignancies and limited therapeutic options. All 47 patients received immune checkpoint inhibition targeting the PD-1 pathway, administered as either nivolumab or pembrolizumab, according to current clinical guidelines. Tumor tissue specimens used for immunohistochemical analyses were obtained either from diagnostic biopsies performed for histopathological confirmation or from surgical tumor resections before starting Anti-PD1 therapy.

Treatment response to immune checkpoint inhibition was assessed by radiologic restaging approximately 8–12 weeks after initiation of therapy using contrast-enhanced computed tomography. Tumor response was evaluated according to the RECIST 1.1 guidelines (23, 24). Patients were classified as having complete response (CR), defined as disappearance of all target lesions; partial response (PR), defined as at least a 30% decrease in the sum of diameters of target lesions; progressive disease (PD), defined as at least a 20% increase in the sum of diameters of target lesions or the appearance of new lesions; or stable disease (SD), defined as neither sufficient shrinkage to qualify for PR nor sufficient increase to qualify for PD.

### HPV tumor status

The HPV tumor status was determined using a combination of p16 immunohistochemical staining and HPV-DNA-PCR analysis. Only those patients who showed both positive p16 IHC staining and positive HPV-DNA-PCR results were classified as having HPV-positive tumors. Due to the notably poorer prognosis and distinct tumor biology observed in discordant cases (where patients tested p16-negative/HPV-DNA-positive or p16-positive/HPV-DNA-negative), it was predefined that both tests need to be positive to assign an HPV-positive tumor status.

For HPV-DNA-PCR analysis, DNA was extracted from tumor samples using the QIAamp DNA Mini Kit (Qiagen, Hilden, Germany) according to the manufacturer’s protocol. The HPV-DNA-PCR was conducted on the LightCycler 2.0 (Roche Diagnostics, Mannheim, Germany) using GP5+/6+ primers, following previously established protocols. Detection of the PCR amplification products was achieved with SYBR Green and gel electrophoresis. The PCR process included an initial denaturation step at 95 °C for 15 min, followed by 45 cycles of denaturation at 95 °C for 10 s, annealing at 45 °C for 5s, and elongation at 72 °C for 18 s. After amplification, a melting curve analysis was performed over a temperature range of 45 °C to 95 °C, with an increase of 0.2 °C per second. Each PCR run included HPV16 and HPV18 positive controls, with melting temperatures (Tm) of 79 °C and 82 °C, respectively. The gene for glyceraldehyde-3-phosphate dehydrogenase (GAPDH) was amplified as an internal control.

For the immunohistochemical detection of p16, the CINtec p16 histology kit (Roche Diagnostics) was used according to the manufacturer’s guidelines on formalin fixed paraffin embedded tissue samples obtained as described below. Epitope retrieval was achieved by heat-induced unmasking after deparaffinization in a rice cooker for 20 min, using the provided retrieval buffer. The p16 antibody was then applied, and the detection of staining was performed as recommended. Each batch of staining included both positive and negative controls.

### Immunohistochemical analysis of TROP2 and TME markers

For immunohistochemical analyses, tumor samples from the primary tumor were available for all 47 patients, obtained either by surgical resection or diagnostic biopsy. In 27 patients, an additional histological sample was collected at the time of locoregional recurrence or distant metastasis. Both primary and recurrent/metastatic tumor specimens were analyzed separately.

Fresh tumor specimens were fixed in 4% PBS-buffered formalin for 24 hours and subsequently embedded in paraffin using the Tissue-Tek^®^ VIP™5 JR tissue processor (Sakura Finetek, Ulm, Germany). After paraffin embedding, three initial 10 μm sections were discarded to avoid surface artifacts. Thereafter, 3 μm-thick sections were prepared using a Leica RM2235 rotary microtome (Leica Microsystems, Wetzlar, Germany). Sections were mounted onto Superfrost Ultra Plus glass slides (Menzel-Gläser, Braunschweig, Germany) and dried overnight at room temperature.

Formalin-fixed, paraffin-embedded (FFPE) tumor tissue samples were used for immunohistochemical (IHC) analysis of TROP2 expression and tumor microenvironment markers, including CD3, CD8, CD4, FOXP3, CCR4, and CD163. These markers were employed as surrogate markers for distinct immune cell populations: CD3 for pan-T cells, CD8 for cytotoxic T lymphocytes, CD4 for helper T cells, FOXP3 for regulatory T cells, CCR4 for Treg/Th2-associated subsets, and CD163 for M2-polarized tumor-associated macrophages. Staining was performed using validated primary antibodies (FOXP3: clone 236A/E7, Abcam, catalog number ab20034, dilution 1:1500, pH 9, antigen retrieval 6 min; CCR4: clone F9A9K, Cell Signaling, catalog number 59945, dilution 1:400, pH 6, antigen retrieval 10 min; CD163: clone EPR19518, Abcam, catalog number ab182422, dilution 1:800, pH 9, antigen retrieval 10 min) according to the manufacturers’ protocols. CD8 (clone C8/144B, Agilent, dilution 1:50); CD4 (clone SP35, Roche, ready-to-use); and CD3 (clone 2GV6, Roche, ready-to-use) immunohistochemical stainings were performed on the BenchMark Ultra IHC/ISH system (Roche, Basel, Switzerland) according to the manufacturer’s standardized protocols. For IHC detection of CD3, CD4 and CD8 the UltraView Universal Alkaline Phosphatase Red Detection Kit (Roche) with Fast Red as chromogen was used. For IHC detection of TROP2, FOXP3, CCR4 and CD163 the Dako REAL Detection System AP/RED (K5005, Agilent) was used. Appropriate positive and negative control tissues were included in each staining run to ensure staining specificity and quality.

### Evaluation of TROP2 expression and immune cell infiltration

A semiquantitative assessment of IHC staining was performed using the Immunoreactivity Score (IRS) as described by Remmele and Stegner (1987) (25). The IRS (range 0–12) was calculated by multiplying staining intensity (0–3) by the percentage of positive tumor resp. immune cells (0–4). Staining intensity was categorized as negative (0), weak (1), moderate (2), or strong (3), while the proportion of positive tumor cells was classified as 0% (0), <10% (1), 10–50% (2), 51–80% (3), or >80% (4).

**Figure 1:**
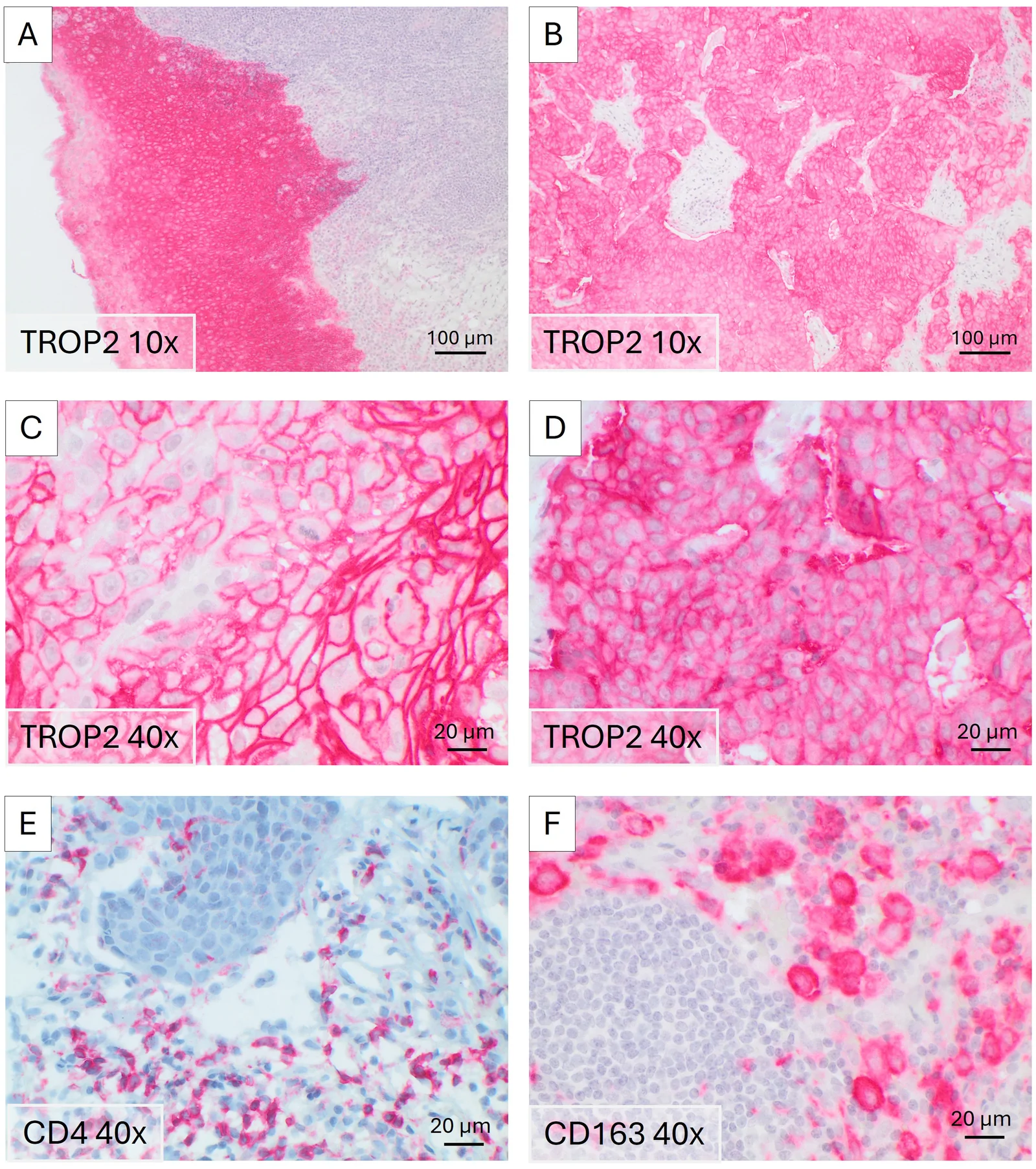
Representative immunohistochemical staining of TROP2 and immune cell markers in primary HNSCC specimens. Representative images were selected from primary tumor samples to illustrate the characteristic staining patterns observed across the cohort (n = 47). **(A, B)** Low-power magnification (10×) demonstrating tumor histoarchitecture and the definition of intratumoral and peritumoral compartments used for immune cell quantification. **(C, D)** High-power magnification showing strong membranous TROP2 expression with additional cytoplasmic staining in tumor cells. **(E)** Representative CD4 immunostaining illustrating lower immune cell density within tumor nests and increased infiltration in the surrounding tumor stroma. **(F)** Representative CD163 immunostaining demonstrating tumor-associated macrophages within the tumor microenvironment.

TROP2 expression was evaluated exclusively in tumor cells, with particular emphasis on membranous and cytoplasmic staining patterns, as illustrated in **Figures 1C, D**.

Non-tumor control tissue consisted of histologically normal squamous mucosa obtained either from healthy individuals undergoing surgery in the corresponding anatomical region or from tumor-adjacent tissue that was confirmed by routine histopathological examination to be free of malignant infiltration. These samples served as non-neoplastic controls for comparison with HNSCC specimens.

For immune cell markers (CD3, CD8, CD4, FOXP3, CCR4, and CD163), immune cell densities were assessed separately in intratumoral and peritumoral compartments. Intratumoral regions were defined as tumor cell nests and the immediately adjacent stromal areas within the tumor boundaries, whereas peritumoral regions comprised stromal tissue located at the invasive tumor front, as depicted in **Figure 1E**.

All stained slides were independently evaluated by three investigators, including a board-certified pathologist, to minimize observer bias. For statistical analyses, the arithmetic mean of the three individual IRS values per sample was used. Expression levels were subsequently dichotomized into high and low expression groups based on the cohort mean IRS value as the cutoff.

To assess inter-observer reliability, all immunohistochemical slides were evaluated independently. Agreement between observers was assessed using the intraclass correlation coefficient (ICC2,k) based on a two-way random-effects model with absolute agreement. The mean IRS of the three observers was used for all subsequent statistical analyses.

### TCGA data analysis

Transcriptomic and clinical data from 483 patients with head and neck squamous cell carcinoma (HNSCC) were obtained from The Cancer Genome Atlas dataset (TCGA-HNSC cohort). Since detailed treatment information is not uniformly available for patients within the TCGA HNSC cohort, overall survival analyses were conducted independently of treatment modality.

Patients were stratified into TROP2-high and TROP2-low groups based on the median TROP2 mRNA expression value. Immune infiltration and immune-related transcriptional programs were quantified using the pan-cancer immune signatures described by Thorsson et al. (26) and derived from established immune deconvolution algorithms. A total of more than 60 immune features (**Supplementary Table 1**) were evaluated, encompassing major adaptive and innate immune cell populations as well as functional immune pathways, including CD8^+^ and CD4^+^ T-cell subsets, follicular helper and γδ T cells, B cells, dendritic cells, monocytes, macrophage subsets (M0 and M2), lymphocyte signatures, Th2 polarization, and TGF-β-associated signaling. Immune signature enrichment scores are reported as normalized Z-scores, representing the number of standard deviations from the mean expression across the TCGA-HNSC cohort.

To account for multiple testing in the TCGA immune signature analysis, nominal p-values were adjusted using the Benjamini–Hochberg false discovery rate (FDR) procedure. Both nominal p-values and FDR-adjusted q-values are reported, with an FDR-adjusted q-value < 0.05 considered statistically significant.

### Statistical analysis

Statistical analyses were performed using GraphPad Prism 9 (GraphPad Software, Boston, MA, USA). Normal distribution was assessed using Anderson-Darling, D’Agostino & Pearson, Shapiro-Wilk, and Kolmogorov–Smirnov tests.

If at least two tests indicated normal distribution, parametric tests were applied (unpaired t-test with Welch’s correction or one-way ANOVA). Otherwise, non-parametric tests (Mann–Whitney U test or Kruskal–Wallis test) were used.

Overall survival (OS), OS since initiation of immunotherapy, and progression-free survival (PFS) were analyzed using the Mantel–Cox (log-rank) test and visualized by Kaplan–Meier curves. For all survival analyses, hazard ratios (HRs) with corresponding 95% confidence intervals (CIs) were calculated and reported in addition to log-rank p-values.

Correlation between TROP2 expression and immune markers was analyzed using Spearman’s rank correlation coefficient (Spearman’s r).

A p-value < 0.05 was considered statistically significant (α = 0.05). The specific statistical tests used are indicated in the figure legends or in the main text, respectively.

## Results

### TROP2 expression and correlation with immune cell infiltration in primary tumors and recurrent/metastatic disease

Inter-observer agreement for IRS scoring among the three independent investigators was good (ICC(2,k)=0.860,95% CI 0.773-0.917), indicating high reproducibility of the semiquantitative immunohistochemical assessment.

**Figure 2:**
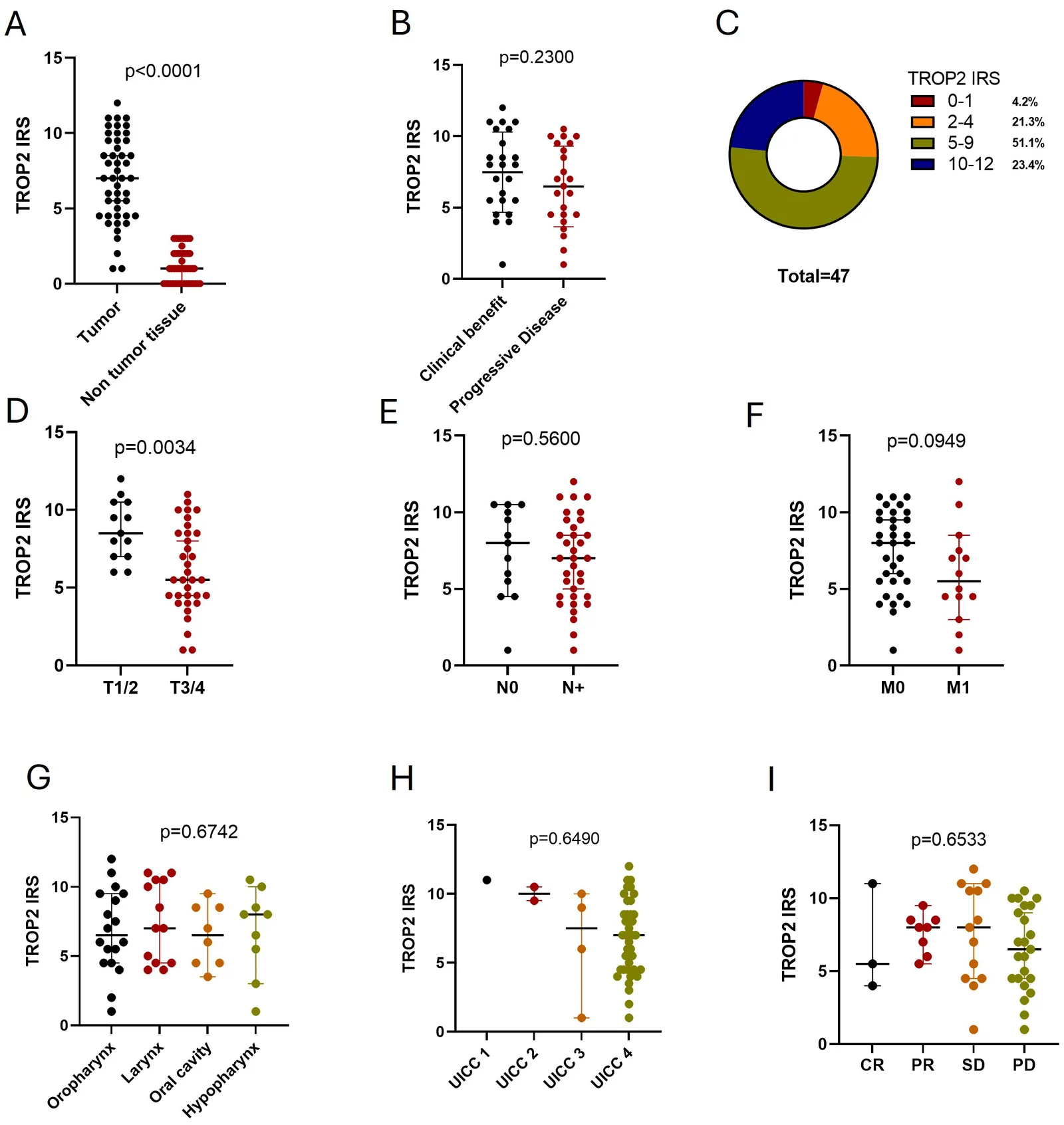
Association of TROP2 expression with clinicopathological characteristics in primary HNSCC (n = 47). **(A)** Representative comparison of TROP2 expression in adjacent non-neoplastic mucosa and tumor tissue. **(B)** Association between TROP2 IRS and response to PD-1 inhibitor therapy categorizing patients into clinical benefit (CR, PR, SD) vs progressive disease. **(C)** Distribution of TROP2 immunoreactivity scores (IRS). **(D–F)** Association between TROP2 IRS and T and N stage. **(F)** Association with M stage. **(G)** Primary tumor localization. **(H)** UICC stage. **(I)** Best radiological response to PD-1 inhibitor therapy according to iRECIST (CR, PR, SD, PD). TROP2 expression was quantified using the IRS. Boxplots display the median, and 95% confidence intervals (CIs). Statistical comparisons were performed using unpaired *t*-test or Mann–Whitney *U* test for two-group comparisons, depending on data distribution, and one-way ANOVA or Kruskal–Wallis tests for comparisons involving more than two groups.

TROP2 expression was highly prevalent in the HNSCC cohort analyzed by IHC (**Figure 1**). Immunohistochemical staining demonstrated predominantly membranous localization, with additional cytoplasmic staining observed to a lesser extent. Overall, more than 95% of tumors showed TROP2 expression (IRS>2), which was highly significant compared with non-tumor tissue (p < 0.0001) (**Figures 2A, C**). The majority of tumors demonstrated moderate to strong expression, with 51.1% exhibiting IRS scores of 5–9 and 23.4% showing strong expression (IRS 10–12). Absent expression was observed in only a minority of cases. Physiological tissue displayed minimal TROP2 staining, primarily in epithelial structures and salivary gland tissue.

Analysis of immune cell populations revealed several associations between TROP2 expression and immune infiltration within the tumor microenvironment (**Supplementary Figures 1–4**). In primary tumors, a significant positive correlation between higher TROP2 IRS and intratumoral FOXP3+ immune cells was observed (p = 0.046, **Supplementary Figure 1**). For other immune cell populations, no statistically significant associations were detected; however, trends toward positive correlations were noted for CCR4+ cells (p = 0.052), CD4+ T cells (p = 0.060), and CD163+ macrophages (p = 0.0504) within the intratumoral compartment.

In the peritumoral compartment, higher TROP2 expression was significantly associated with increased immune infiltration with CD163+ macrophages (p = 0.0084), CCR4+ cells (p = 0.043), and FOXP3+ regulatory T cells (p = 0.031).

In contrast, analysis of recurrent or metastatic (R/M) disease cases demonstrated a different pattern. Here, higher TROP2 expression was significantly associated with lower CD3+ T-cell infiltration, both intratumorally (p = 0.018) and peritumorally (p = 0.024). No significant associations were observed between TROP2 expression and other immune cell populations in the R/M tumor microenvironment. A trend toward higher TROP2 IRS values was observed in patients with clinical benefit (CR, PR,SD) compared with those with progressive disease; however, the difference was not statistically significant (p = 0.23, **Figure 2B**).

**Figure 3:**
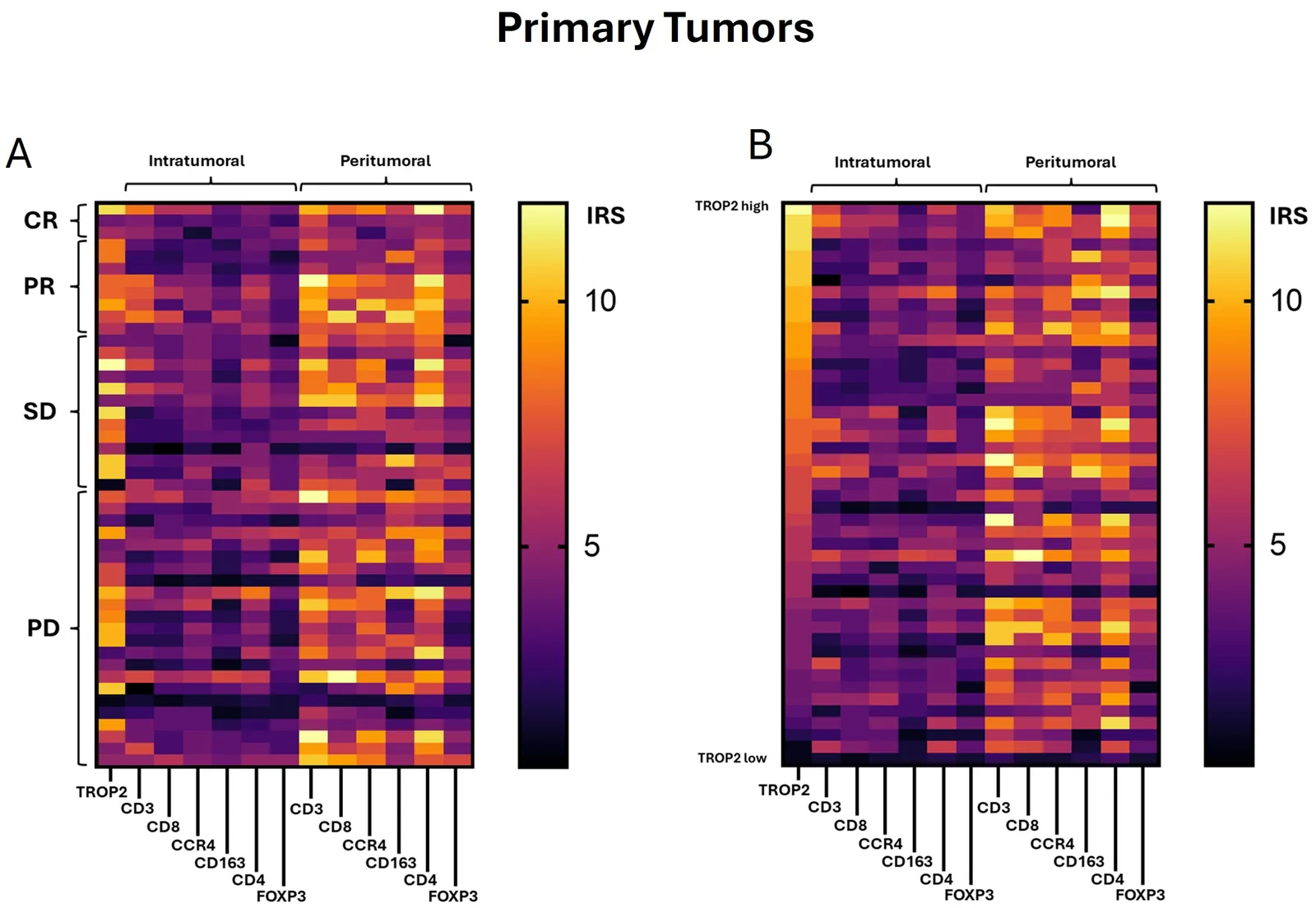
Heatmap analysis of TROP2 expression and immune cell infiltration in primary HNSCC specimens (n = 47). Heatmaps display TROP2 immunoreactivity scores (IRS) together with intratumoral and peritumoral immune cell infiltration (CD3, CD8, CD4, FOXP3, CCR4, and CD163). IRS values are shown using a color gradient, with darker colors indicating lower expression and brighter colors indicating higher expression. **(A)** Samples clustered according to radiological response to PD-1 inhibitor therapy (complete response [CR], partial response [PR], stable disease [SD], progressive disease [PD]). **(B)** Samples ordered according to decreasing TROP2 IRS to visualize the relationship between TROP2 expression and immune cell infiltration. No hierarchical clustering was applied.

Visualization of these relationships in the heatmap (**Figures 3A, B**) further illustrates that overall immune infiltration was generally higher in the peritumoral compartment compared with the intratumoral compartment. Stratification according to response to immunotherapy [complete response (CR), partial response (PR), stable disease (SD), and progressive disease (PD)] did not reveal a clear association with TROP2 expression.

Evaluation of TROP2 expression across primary tumor sites demonstrated no significant differences between oropharyngeal, laryngeal, oral cavity, and hypopharyngeal carcinomas (p = 0.67). Notably, lower tumor stages (T1/2) showed significantly higher TROP2 expression compared with advanced stages (T3/4). In contrast, N status and M status showed similar trends but did not reach statistical significance (**Figures 2D–F**).

Furthermore, TROP2 expression did not significantly differ between UICC stage or response to immunotherapy.

### Survival outcomes and associations with TROP2 expression and immune cell infiltration

**Figure 4:**
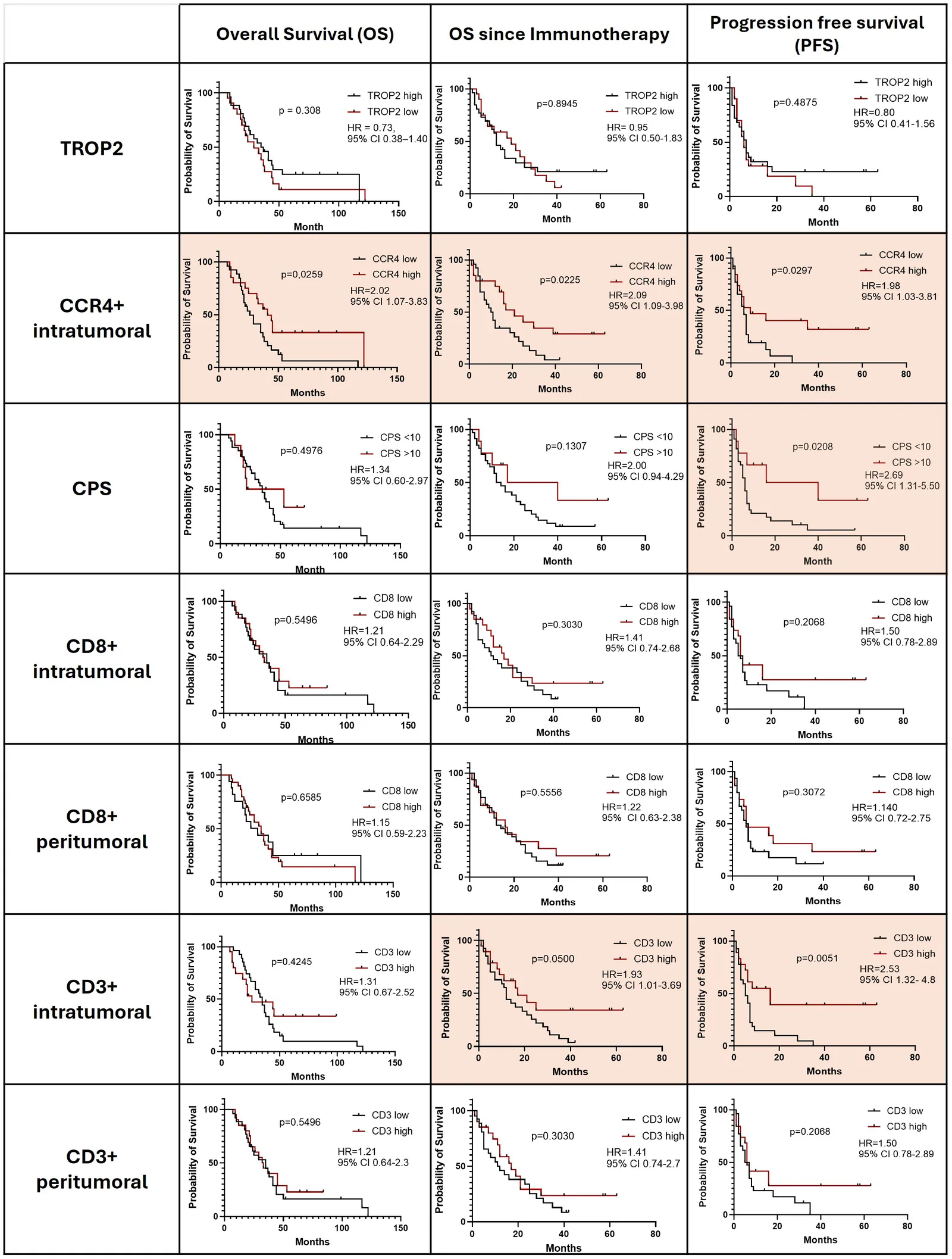
Kaplan–Meier survival analyses according to TROP2 expression and immune cell infiltration. Kaplan–Meier curves depict overall survival (OS), overall survival from initiation of immunotherapy (OS since IT), and progression-free survival (PFS). High and low expression groups were defined using the cohort mean IRS as the cut-off. Hazard ratios (HRs), 95% confidence intervals (CIs) and log-rank (Mantel–Cox) *p*-values are shown for each analysis. Censored observations are indicated by tick marks. Statistically significant associations are highlighted in orange.

Survival analyses were performed for overall survival (OS) since first tumor diagnosis, overall survival since initiation of immunotherapy (OS since IT), and progression-free survival (PFS). The median follow-up time was 44 months. No statistically significant association was observed between TROP2 expression status and any of the evaluated survival endpoints. However, Kaplan–Meier curves demonstrated a trend toward improved survival outcomes in patients with higher TROP2 expression (**Figure 4**).

In contrast, higher intratumoral CCR4+ immune cell infiltration was significantly associated with improved survival outcomes, including OS, OS since IT, and PFS.

Patients with PD-L1 CPS >10 demonstrated trends toward improved OS and OS since IT, while a significant improvement was observed for PFS (p = 0.02).

Analysis of CD8+ T-cell infiltration revealed trends toward improved OS and PFS in patients with higher CD8+ cell densities in both intratumoral and peritumoral compartments, although these differences did not reach statistical significance.

Interestingly, high intratumoral CD3+ T-cell infiltration was significantly associated with improved OS since immunotherapy (p = 0.05) and PFS (p = 0.0051). In contrast, peritumoral CD3+ infiltration did not show a significant association with survival outcomes.

Additional Kaplan–Meier survival analyses for clinicopathologic and immune cell parameters are presented in the **Supplementary Figures**. These analyses largely confirmed the trends observed in the main results, although statistical significance was not consistently reached, likely reflecting the limited sample size (**Supplementary Figures 1**, **2**).

### TROP2 mRNA expression and correlation with immune features in the TCGA HNSC cohort

To further investigate the relationship between TROP2 and the tumor immune microenvironment, transcriptomic data from the TCGA HNSC cohort (n = 483) were analyzed. Patients were stratified according to the median of TROP2 mRNA expression levels, and immune signatures were compared between TROP2-high and TROP2-low tumors. As comprehensive treatment information, including the administration of immune checkpoint inhibitors, is not uniformly available within the TCGA cohort, all survival analyses were performed independently of treatment modality and should therefore be interpreted as prognostic rather than predictive.

**Figure 5:**
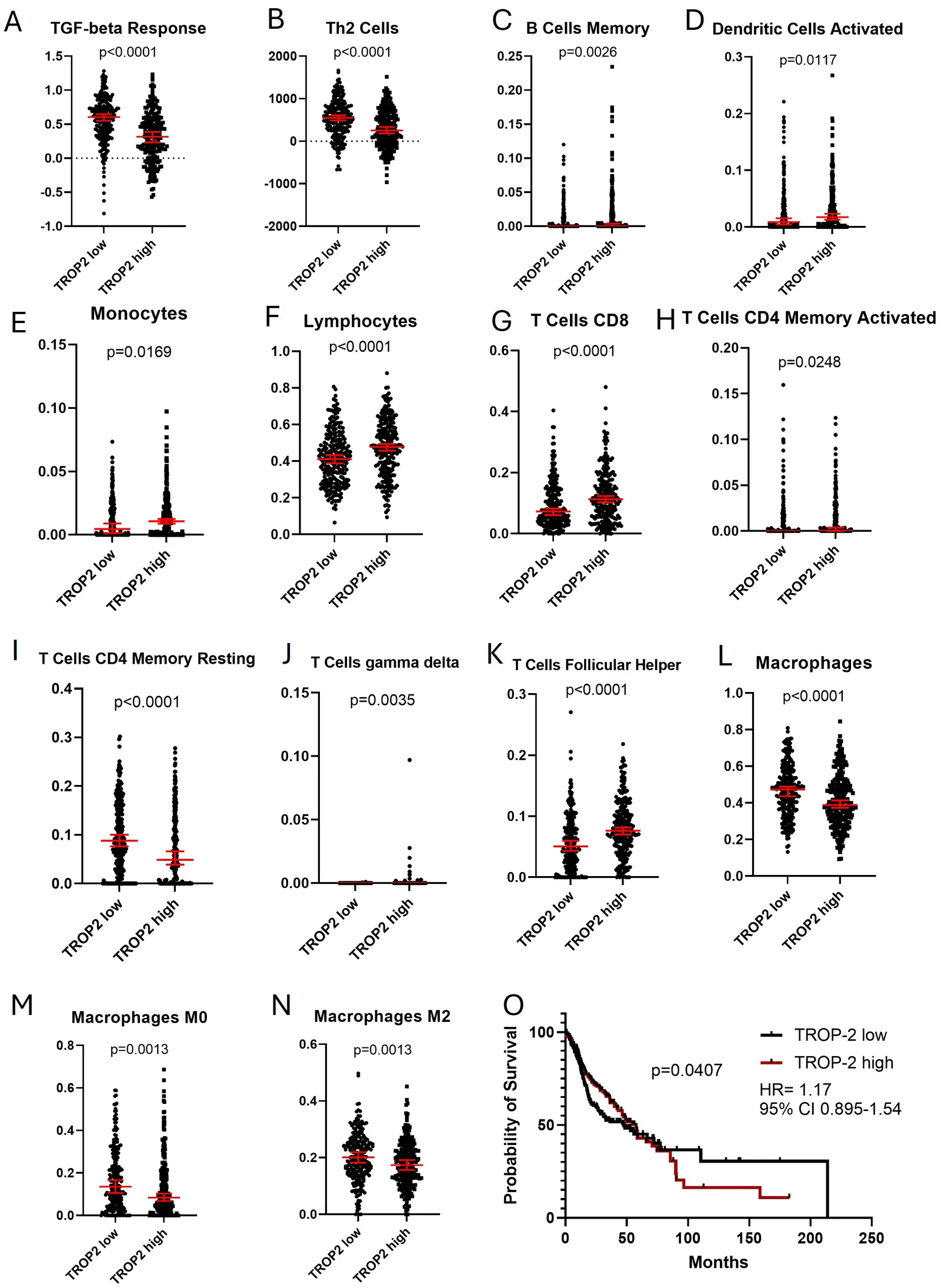
TROP2 mRNA expression and immune-related transcriptional signatures in the TCGA-HNSC cohort (n = 483). **(A–N)** Comparison of immune cell populations and immune-related gene signatures between tumors with low and high TROP2 mRNA expression, dichotomized using the cohort median. Gene expression values are presented as normalized Z-scores relative to the TCGA cohort mean. Statistical comparisons were performed using the Mann -Whitney U test. Nominal p-values are shown in the image, while corresponding Benjamini -Hochberg false discovery rate (FDR)-adjusted q-values are provided in **Supplementary Table 2**. **(O)** Kaplan -Meier analysis demonstrating overall survival according to TROP2 mRNA expression. HRs, 95% CIs, log-rank p-value are shown.

To account for multiple testing, false discovery rate (FDR) correction using the Benjamini–Hochberg procedure was applied. Following FDR adjustment, the associations of monocytes and activated CD4^+^ memory T cells with TROP2 expression no longer remained statistically significant (q > 0.05, **Figure 5**), whereas all other reported associations retained statistical significance (**Supplementary Table 2**).

A complex and context-dependent relationship between TROP2 expression and the tumor immune microenvironment was observed. Several immune cell populations and immune-related pathways showed significant associations with TROP2 expression. Tumors with high TROP2 expression demonstrated significantly increased enrichment of monocytes, CD8+ T cells, Lymphocytes, follicular helper T cells, Gamma delta (γδ) T cells, memory B cells and activated dendritic cells, In contrast, TGF-β response pathways, Th2 cells, CD4+ memory T cells (resting), macrophages (M0 and M2) were inversely correlated with TROP2 expression, indicating a differential regulation of immunosuppressive signaling pathways.

Notably, survival analyses of the TCGA HNSC cohort demonstrated that patients with high TROP2 mRNA expression exhibited significantly different overall survival compared with patients with low TROP2 expression. Given the absence of standardized treatment information within TCGA, this finding reflects the prognostic association of TROP2 expression rather than its predictive value for response to specific treatments.

Overall, these findings suggest that TROP2 expression is associated with a distinct immune-related transcriptional landscape in HNSCC, characterized by enrichment of multiple adaptive and innate immune cell populations.

### TROP2 expression and tumor microenvironment in recurrent and metastatic disease

**Figure 6:**
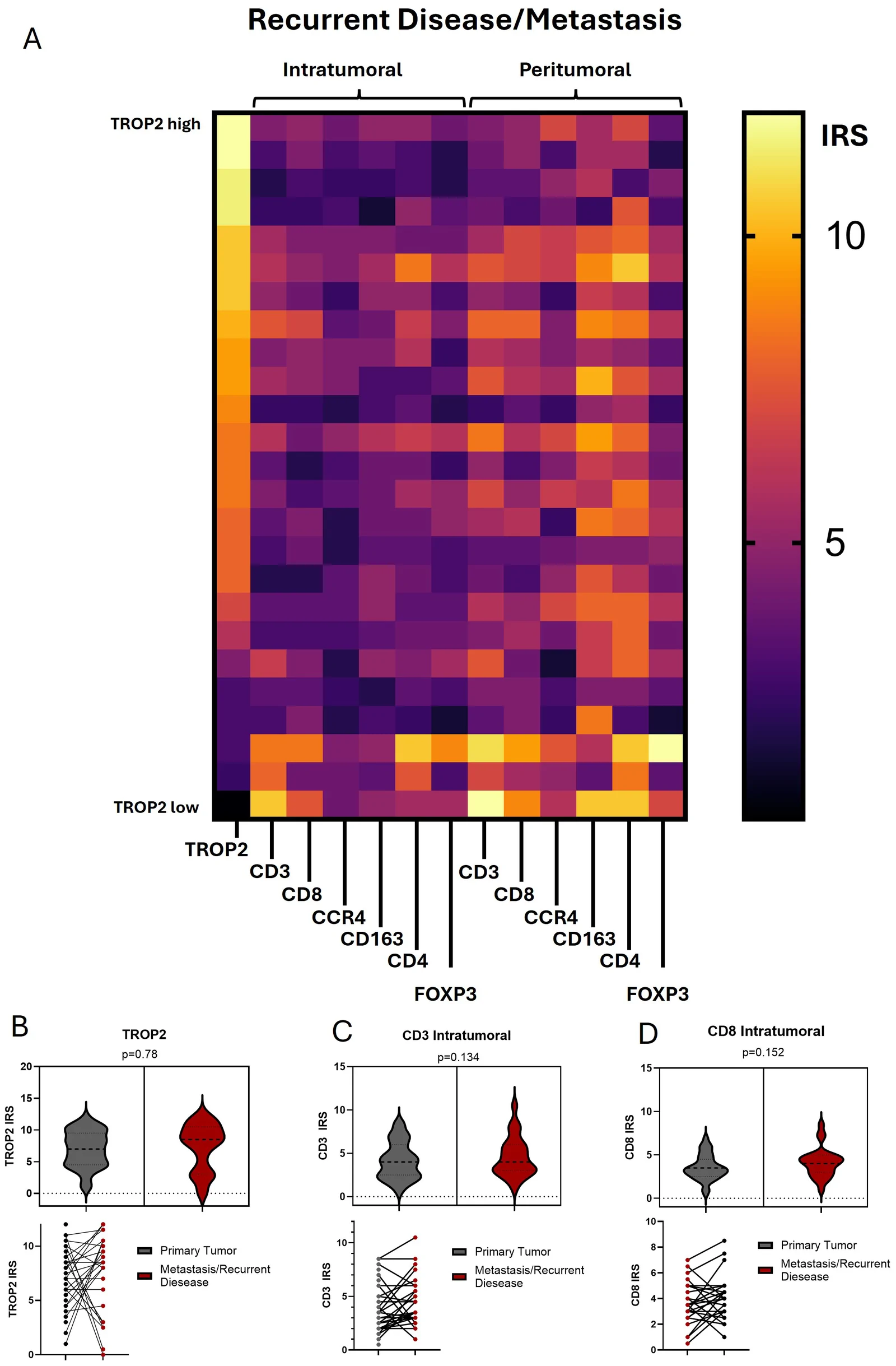
TROP2 expression and immune cell infiltration in paired recurrent/metastatic HNSCC specimens. Heatmap visualization of TROP2 IRS and intratumoral/peritumoral immune cell infiltration (CD3, CD8, CD4, FOXP3, CCR4, CD163) in recurrent and metastatic specimens obtained from 27 patients with matched primary and recurrent/metastatic tissue samples. IRS values are displayed using a color scale from low (dark) to high (bright) expression or immune cell density. Samples are ordered according to decreasing TROP2 IRS. **(B–D)** Paired slope plots comparing TROP2 IRS, intratumoral CD3^+^ T-cell infiltration, and intratumoral CD8^+^ T-cell infiltration between matched primary and recurrent/metastatic specimens. Each line represents one patient. Violin plots are shown alongside the paired analyses to visualize the distribution of the respective measurements in primary and recurrent/metastatic tissues. Statistical comparisons were performed using the Wilcoxon matched-pairs signed-rank test.

In 27 of the 47 patients, additional tumor samples from locoregional recurrence or distant metastasis were available and analyzed to further explore the relationship between TROP2 expression and the tumor microenvironment in recurrent/metastatic disease (see **Table 2**). Heatmap visualization demonstrated heterogeneous immune cell infiltration patterns across the analyzed specimens, while TROP2 expression levels are shown on the left side of the heatmap for comparison (**Figure 6**).

**Table 2:** Overview of the study cohort and tissue sample availability.

Parameter	Number of patients (%)
Total patients included	47 (100%)
Primary tumor samples available	47 (100%)
Patients with additional recurrence/metastasis samples	27 (57.4%)
– Locoregional recurrence	20 (42.6%)
– Distant metastasis	7 (14.9%)
—— Pulmonary metastasis	5 (10.6%)
—— Hepatic metastasis	2 (4.3%)
Patients without additional sampling	20 (42.6%)

Primary tumor specimens were available for all patients. In a subset of patients, additional tissue samples were obtained at the time of locoregional recurrence or distant metastasis and included in the analysis.

Overall, the analysis indicated that immune cell infiltration remained predominantly higher in the peritumoral compartment compared with the intratumoral compartment, consistent with our observations in primary tumors. However, in contrast to primary tumor samples, higher TROP2 expression in R/M disease showed a tendency toward reduced intratumoral immune cell infiltration, particularly within the CD3+ T-cell population, which was significantly decreased in tumors with high TROP2 expression.

Other immune cell populations, including CD8+ T cells, CD4+ T cells, FOXP3+ regulatory T cells, CCR4+ cells, and CD163+ macrophages, demonstrated heterogeneous patterns without consistent significant correlations with TROP2 expression in the recurrent/metastatic setting.

These findings suggest that the relationship between TROP2 expression and immune cell composition may change during disease progression, potentially reflecting alterations in tumor–immune interactions in advanced HNSCC.

Paired analysis of primary and recurrent/metastatic specimens was performed in the subgroup of 27 patients with matched tissue samples to assess longitudinal changes in TROP2 expression and immune cell infiltration. TROP2 expression remained stable between primary tumors and the corresponding recurrent/metastatic lesions, with no significant difference in IRS (p=0.78). In contrast, recurrent/metastatic specimens showed a trend toward increased intratumoral CD3^+^ T-cell infiltration (p = 0.134) and CD8^+^ cytotoxic T-cell infiltration (p = 0.152), although these differences did not reach statistical significance (**Figures 6B–D**).

## Discussion

In this study, we investigated TROP2 expression and its association with the tumor immune microenvironment in a cohort of 47 patients with HNSCCs treated with anti-PD1 immunotherapy, including both primary tumors and in 27 cases matched recurrent/metastatic disease. Using immunohistochemistry and complementary transcriptomic analyses from The Cancer Genome Atlas, we evaluated TROP2 expression patterns, immune cell infiltration, and their relationship with clinicopathologic parameters, response to PD-1 blockade, and survival outcomes.

Overall, TROP2 was highly expressed in the majority of tumors and was associated with distinct immune infiltration patterns, including correlations with regulatory and myeloid immune cell populations. While TROP2 expression did not significantly correlate with response to immunotherapy or survival in our cohort, immune cell infiltration particularly CCR4+ and CD3+ T cells showed stronger prognostic relevance. These findings reinforce previous observations that TROP2 is broadly expressed across epithelial malignancies (27–29) and may represent a stable tumor-associated surface antigen suitable for targeted therapeutic strategies (19, 30–32).

In HNSCC and other squamous cell carcinomas, TROP2 expression appears to reflect the epithelial lineage, shaping the tumor microenvironment and differentiation state of tumor cells (33), rather than acting solely as a marker of aggressive tumor biology, while reduced expression has been observed in poorly differentiated squamous cell carcinomas and during tumor progression (12, 34, 35). Consistent with this concept, our analysis demonstrated significantly higher TROP2 expression in early T-stage tumors (T1–T2) compared with advanced tumors. This observation may indicate that TROP2 expression is preserved during early tumor development and may decrease as tumors acquire more dedifferentiated or mesenchymal phenotypes during progression.

Importantly, we observed no significant differences in TROP2 expression between primary tumors and recurrent or metastatic lesions, suggesting that TROP2 expression remains relatively stable throughout disease evolution. From a therapeutic perspective, this stability is highly relevant because targetable antigens that persist across disease stages are essential for the effective development of antibody-based therapies, including monoclonal antibodies and antibody–drug conjugates (ADCs) as it is already an important therapeutic target in various solid tumors as lung cancer and breast cancer (17, 36–38). The high prevalence and stable expression pattern observed in our cohort therefore further supports TROP2 as a robust therapeutic target in HNSCCs.

A further major focus of the present study was the relationship between TROP2 expression and the immunological TME. In primary tumors, higher TROP2 expression was significantly associated with increased intratumoral FOXP3+ regulatory T cells, peritumoral CD163+ macrophages, CCR4+ immune cells, and FOXP3+ regulatory T cells.

Such immune compositions are well recognized in HNSCC and may contribute to immune escape mechanisms and tumor progression. Tumor-associated macrophages, particularly those expressing CD163, are frequently associated with M2-like polarization and immunosuppressive cytokine production, while FOXP3+ regulatory T cells play a central role in suppressing cytotoxic T-cell responses and maintaining immune tolerance within the tumor microenvironment (39, 40). These findings suggest that TROP2 expression may be linked to an immune-regulatory or immunosuppressive microenvironment, characterized by macrophage polarization and regulatory T-cell recruitment.

Emerging evidence from multiple tumor entities suggests that TROP2 expression is not only linked to tumor-intrinsic signaling but also to modulation of the tumor microenvironment. In NSCLC, urothelial carcinoma and breast cancer, TROP2 overexpression has been associated with poor prognosis, altered immune infiltration patterns, including increased macrophage recruitment and immune evasion mechanisms (41–44). In gastric cancers, TROP2 has been shown to induce multidrug resistance by regulating Notch1 signaling pathway, which is involved in maintaining tissue homeostasis and stem cell regulation (45). Notably, TROP2-targeted CAR-T cell therapy has been shown to eradicate drug-tolerant persister cells in EGFR-mutated non-small cell lung cancer, highlighting a potential role of TROP2 in tumor cell plasticity and therapy resistance (46).

Interestingly, a distinct pattern emerged in recurrent or metastatic tumors, where higher TROP2 expression was associated with significantly lower intratumoral and peritumoral CD3+ T-cell infiltration. This finding suggests that immune contexture may evolve during disease progression, potentially reflecting tumor-driven immune editing or therapy-induced immune remodeling. Such dynamic changes in immune composition have been reported in advanced HNSCC, where tumors may shift toward more immunologically “cold” phenotypes characterized by reduced T-cell infiltration and increased immunosuppressive signaling (47–49). These observations highlight the complexity of tumor–immune interactions and underscore the importance of evaluating both tumor-intrinsic and microenvironmental factors when assessing potential therapeutic targets.

To further validate our findings, we analyzed transcriptomic data from the HNSC cohort of The Cancer Genome Atlas. High TROP2 mRNA expression was significantly associated with multiple immune-related signatures, including CD8+ T cells, CD4+ memory T cells, follicular helper T cells, γδ T cells, dendritic cells, macrophages, and Th2-associated immune responses, as well as TGF-β signaling pathways. These associations suggest that TROP2 expression is embedded within broader immune regulatory networks in the tumor microenvironment. The coexistence of cytotoxic and regulatory immune signatures further highlights the complex and context-dependent immune landscape of HNSCC.

In the TCGA cohort, high TROP2 expression was also associated with reduced overall survival, indicating a potential prognostic role at the transcriptomic level. Although survival analyses in our IHC cohort did not reach statistical significance, Kaplan–Meier curves consistently showed trends toward improved overall and progression-free survival in tumors with higher TROP2 expression. Notably, immune infiltration—particularly intratumoral CCR4+ and CD3+T cells—demonstrated stronger associations with survival outcomes, underscoring the prognostic relevance of immune contexture in HNSCC, as previously reported (39, 50). Owing to the limited sample size and number of survival events, multivariable Cox regression analyses were not performed, as adjustment for multiple clinicopathological covariates would have resulted in overfitting and unreliable effect estimates. Consequently, the survival analyses should be regarded as exploratory and hypothesis-generating.

An apparent discrepancy was observed between the IHC and TCGA survival analyses. While higher TROP2 protein expression showed a non-significant trend toward improved survival in our immunotherapy-treated cohort, elevated TROP2 mRNA expression was associated with significantly poorer overall survival in the TCGA cohort. This difference likely reflects the distinct clinical contexts of the two cohorts. The TCGA analysis represents the prognostic impact of TROP2 in predominantly treatment-naïve HNSCC, whereas our cohort exclusively comprised patients receiving PD-1 blockade in the recurrent/metastatic setting, in whom TROP2 expression may instead reflect differential sensitivity to immunotherapy. Moreover, protein and mRNA expression levels are not necessarily directly comparable due to post-transcriptional and post-translational regulation. These findings underscore the need for prospective studies evaluating TROP2 as both a prognostic and predictive biomarker in HNSCC.

From a translational perspective, the high prevalence and stable expression of TROP2 across HNSCC tumors may have important therapeutic implications. TROP2 has emerged as a clinically actionable target through the development of TROP2-directed antibody–drug conjugates, most notably sacituzumab govitecan, which has demonstrated significant clinical benefit in several epithelial malignancies including metastatic triple-negative breast cancer, NSCLC and urothelial carcinoma (46, 51–54). These ADCs deliver cytotoxic payloads selectively to TROP2-expressing tumor cells and may additionally exert bystander killing effects within the tumor microenvironment. TROP2-directed therapies are increasingly being explored in HNSCC, including phase II trials of Sacituzumab govitecan-based combinations (NCT07063212) and next-generation ADCs such as sacituzumab tirumotecan (NCT07088211), as well as basket studies including HNSCC expansion cohorts, supporting the feasibility of TROP2-targeted approaches in this disease (55).

Importantly, emerging evidence suggests that ADCs may also exert immune-modulatory effects, including the induction of immunogenic cell death, increased antigen presentation, and enhanced dendritic cell activation (56). Such mechanisms raise the possibility that TROP2-targeted ADCs could synergize with immune checkpoint inhibition, particularly therapies targeting PD-1. In HNSCC, PD-1 blockade has already demonstrated clinical efficacy, most notably in the phase III CheckMate 141 trial and the phase III studies KEYNOTE 48 and KEYNOTE-040, which established the anti–PD-1 antibody Nivolumab/Pembrolizumab as an effective therapy for recurrent or metastatic disease (5–7). More recently, the phase III KEYNOTE-689 trial further extended the benefit of PD-1 inhibition into the perioperative setting in locally advanced disease (8, 57). Given the widespread expression of TROP2 in HNSCC and its associations with immune-related pathways, combination strategies integrating TROP2-targeted ADCs with PD-1/PD-L1 inhibitors may represent a promising therapeutic approach. Such strategies could potentially enhance tumor cell killing while simultaneously promoting antitumor immune responses, thereby overcoming immune resistance mechanisms through the conversion of immunologically “cold” tumors into immune-infiltrated (“hot”) tumors (49).

From a critical perspective, several limitations should be acknowledged. The retrospective design and relatively small cohort size may limit statistical power, while clinical heterogeneity in treatment modalities could have influenced survival outcomes and immune microenvironment composition. In addition, although all patients received PD-1 inhibitor therapy in the recurrent/metastatic palliative setting according to current treatment guidelines, the cohort included patients with different initial tumor stages as well as paired primary and recurrent/metastatic tumor specimens. While this reflects real-world clinical practice, it may have contributed to biological heterogeneity and should be considered when interpreting the results. Therefore, larger prospective studies with more homogeneous patient populations are warranted to validate our findings and further define the role of TROP2 in HNSCC. Furthermore the semiquantitative assessment of immune infiltration by immunohistochemistry does not fully capture the spatial and functional complexity of the tumor immune landscape. Although validation using data from The Cancer Genome Atlas strengthens our findings, mRNA expression may not directly reflect protein expression or functional activity, and mechanistic experiments investigating causal links between TROP2 signaling and immune regulation were beyond the scope of this study. Another limitation of this study is the use of cohort-dependent cut-offs for TROP2 expression, as no validated threshold currently exists for HNSCC. While the mean IRS (IHC cohort) and median mRNA expression (TCGA cohort) are established approaches for exploratory analyses, they may limit the generalizability of the findings.

Despite these limitations, our results provide integrated protein-level and transcriptomic evidence linking TROP2 expression to immune microenvironment features in HNSCC. The high prevalence of TROP2 expression and its association with immune signatures support further investigation of TROP2 as both a biomarker and therapeutic target, particularly in strategies combining TROP2-targeted antibody–drug conjugates with PD-1/PD-L1 checkpoint inhibition.

## Data Availability

The datasets presented in this study can be found in online repositories. The names of the repository/repositories and accession number(s) can be found in the article/**Supplementary Material**.
