# Supplementary material for: TROP2 expression in head and neck squamous cell carcinoma: association with the tumor immune microenvironment and clinical outcome
Source: Front Oncol. 2026 Aug 6;16:1901872. doi: 10.3389/fonc.2026.1901872 (PMC13489803; doi:10.3389/fonc.2026.1901872)
Supplement: Supplementary file 1 [file DataSheet1.docx]

**Supplementary Figure 1:** Spearman correlation analysis demonstrating the relationship between intratumoral TROP2 expression and immune cell populations in primary tumors. Correlation coefficients (Spearman’s r) are shown for CD3⁺, CD8⁺, CD4⁺, FOXP3⁺, CCR4⁺, and CD163⁺ immune cells and for CPS and TPS.

**Supplementary Figure 2**: Spearman correlation analysis demonstrating the relationship between peritumoral TROP2 expression and immune cell populations in primary tumors. Correlation coefficients (Spearman’s r) are shown.

**Supplementary Figure 3:** Spearman correlation analysis demonstrating the relationship between TROP2 expression and intratumoral immune cell populations in recurrent and/or metastatic tumors. Correlation coefficients (Spearman’s *r*) are shown, illustrating the association between TROP2 expression and immune cell infiltration in the recurrent/metastatic tumor microenvironment.

**Supplementary Figure 4:** Spearman correlation analysis demonstrating the relationship between TROP2 expression and immune cell populations in recurrent and/or metastatic tumors. Correlation coefficients (Spearman’s *r*) are shown, illustrating the association between TROP2 expression and immune cell infiltration in the recurrent/metastatic peritumoral tumor microenvironment.

**Supplementary Figure 5:** Kaplan–Meier survival analyses illustrating overall survival (OS), overall survival since initiation of immunotherapy, and progression-free survival (PFS) according to intratumoral or peritumoral immune cell infiltration in HNSCCs. **Supplementary Figure 6:** Kaplan–Meier survival analyses illustrating overall survival (OS), overall survival since initiation of immunotherapy, and progression-free survival (PFS) according to intratumoral or peritumoral immune cell infiltration in HNSCCs.
